# Response of litter decomposition and the soil environment to one-year nitrogen addition in a Schrenk spruce forest in the Tianshan Mountains, China

**DOI:** 10.1038/s41598-021-04623-8

**Published:** 2022-01-13

**Authors:** Zhaolong Ding, Xu Liu, Lu Gong, Xin Chen, Jingjing Zhao, Wenjing Chen

**Affiliations:** 1 College of Resources and Environment Science, Xinjiang University, Urumqi, 830046 China; 2 Key Laboratory of Oasis Ecology, Xinjiang University, Urumqi, 830046 China; 3 State Key Laboratory of Grassland and Agro-Ecosystems, School of Life Sciences, Lanzhou University, 222 Tianshui Road, Lanzhou, 730000 Gansu China

**Keywords:** Forest ecology, Forestry

## Abstract

Human activities have increased the input of nitrogen (N) to forest ecosystems and have greatly affected litter decomposition and the soil environment. But differences in forests with different nitrogen deposition backgrounds. To better understand the response of litter decomposition and soil environment of N-limited forest to nitrogen deposition. We established an in situ experiment to simulate the effects of N deposition on soil and litter ecosystem processes in a *Picea schrenkiana* forest in the Tianshan Mountains, China. This study included four N treatments: control (no N addition), low N addition (LN: 5 kg N ha^−1^ a^−1^), medium N addition (MN: 10 kg N ha^−1^ a^−1^) and high N addition (HN: 20 kg N ha^−1^ a^−1^). Our results showed that N addition had a significant effect on litter decomposition and the soil environment. Litter mass loss in the LN treatment and in the MN treatment was significantly higher than that in the control treatment. In contrast, the amount of litter lost in the HN treatment was significantly lower than the other treatments. N application inhibited the degradation of lignin but promoted the breakdown of cellulose. The carbon (C), N, and phosphorus (P) contents of litter did not differ significantly among the treatments, but LN promoted the release of C and P. Our results also showed that soil pH decreased with increasing nitrogen application rates, while soil enzyme activity showed the opposite trend. In addition, the results of redundancy analysis (RDA) and correlation analyses showed that the soil environment was closely related to litter decomposition. Soil enzymes had a positive effect on litter decomposition rates, and N addition amplified these correlations. Our study confirmed that N application had effects on litter decomposition and the soil environment in a N-limited *P. schrenkiana* forest. LN had a strong positive effect on litter decomposition and the soil environment, while HN was significantly negative. Therefore, increased N deposition may have a negative effect on material cycling of similar forest ecosystems in the near future.

## Introduction

The litter of forest ecosystems is rich in nutrients and organic matter. Litter decomposition is a key ecological process for energy flow and material circulation in forest ecosystems and it plays an important role in maintaining the nutrient balance both aboveground and belowground^1–3^. The litter decomposition process is affected by biotic and abiotic factors, including global change, litter substrates, decomposers, and the soil environment^4–6^. At present, increase in nitrogen (N) deposition caused by human activities (e.g., industry, agriculture, and husbandry) has become an important driver of global change, while deposition rates are likely to continue increasing in the future^7,8^. Increasing N deposition has led to a continuous increase in N input in terrestrial ecosystems^9^. Forests are the main recipients of nitrogen deposition in terrestrial ecosystems^10^. Increased N deposition has also affected litter decomposition dynamics^11,12^. In forest ecosystems, studying the response of litter decomposition and the soil environment to N addition is crucial for understanding and predict forest ecosystem responses and adaptations to long-term N deposition. In the past decades, this need has prompted extensive research on litter decomposition under significantly increased N deposition.

To explore the effect of increased nitrogen deposition on litter decomposition, experiments on simulated N deposition have been conducted. Increasing evidence shows that N plays an important role in litter decomposition, whether the N is derived from the litter itself or from environmental sources^12^. However, there is little evidence in the current literature that N addition has a strong directional effect on litter decomposition as it may have a positive, negative or no effect. For example, Hobbie and Vitousek (2000) observed that N addition promoted the decomposition of litter^13^, while Zhou et al. found that N addition inhibited the decomposition of evergreen broad-leaved forest litter in western China^14^. In addition, Wang et al. found that N addition had no effect on the litter decomposition of *Populus tremuloides* in a boreal forest^15^. Some studies suggested that due to regional differences in N deposition and differences in forest types and fertilization levels, N addition inhibits litter decomposition in N-saturated tropical forest areas^16^ but promotes litter decomposition in N-deficient temperate forests at low levels of N addition^17^. At high levels of N addition, even these latter forests show reduced litter decomposition^12^. At the same time, the different litter substrate characteristics of different forest plants (e.g., lignin, cellulose, lignin: N, C:N:P ratios) determine the decomposability and nutrient utilization of litter^5,18,19^, leading to different responses of litter decomposition to N addition. Some studies have found that N addition promotes litter decomposition with low lignin content and slows litter decomposition with high lignin content^20^. Other studies have also reported that high levels of N addition inhibit the degradation of lignin and cellulose, suppressing litter decomposition, while smaller N additions promote the degradation of lignin and cellulose^21^. In addition, nutrient release and accumulation are an important part of the nutrient cycle of forest ecosystems. These processes occur simultaneously with litter decomposition, are closely related to the dynamic changes in litter decomposition and are affected by the increase in N deposition^22,23^. Therefore, the response of litter decomposition dynamics to N addition needs to be further investigated in different forest ecosystems, especially in controversial N-limited forests.

N addition also changes the biological and abiotic characteristics of the soil and their interactions, including soil pH, soil nutrients, and soil biological characteristics^24–26^. Most studies have shown that N addition decreased soil pH, resulting in soil acidification^25^. N addition will also increase the availability of soil N and alter the balance between soil C and P, such as lowering the C:N ratios and increasing the N:P ratios, thereby reducing available C and increasing the loss of P^26,27^. Enzymes are important catalytic factors in the litter decomposition process. However, the effect of nitrogen addition on enzyme activity is controversial. Some studies have shown that N addition inhibited the activities of phosphatase, phenol oxidase, lignin- and cellulose-degrading enzymes and promoted glycosidase activity^24^. The soil environment significantly affects the litter decomposition process. However, there are still many unknowns about how soil chemical and biological characteristics change and what role they play in litter decomposition under different N additions. Therefore, studying changes in the soil environment with N addition can improve our understanding of the response of litter decomposition to N addition.

China has become one of the top three regions of the world for high rates of N deposition^28^. Although the level of N deposition in western China is lower than the national average, it is increasing every year^9,29^. The Tianshan Mountain Range is the largest arid mountain system in the world and is located in western China. *Picea schrenkiana* forests are the main forest ecosystem in the Tianshan Mountains. It plays an irreplaceable role in soil and water conservation, carbon fixation and oxygen release, maintaining biodiversity and ecosystem stability throughout arid parts of western China^30–32^. Litter in the *P. schrenkiana* forests of the Tianshan Mountains is characterized by its low quality and high C:N ratios^33^ ratios. Previous studies on *Picea schrenkiana* forests include the response of *Picea schrenkiana* roots to N addition^34,35^ and the effect of snow cover on litter decomposition^33^. The *Picea schrenkiana* forest is also affected by increases in regional N deposition due to the intensification of human activities. Therefore, we conducted a one-year simulated N deposition to explore how the litter decomposition process and soil environment responded. Our purpose was to (1) evaluate the degree and directional change of litter decomposition in *Picea schrenkiana* forests and the response of the soil environment to N addition, as well as to (2) determine the relationship between litter decomposition and the soil environment under N addition.

## Results

### Effects of N addition on litter decomposition dynamics

**Figure 1:**
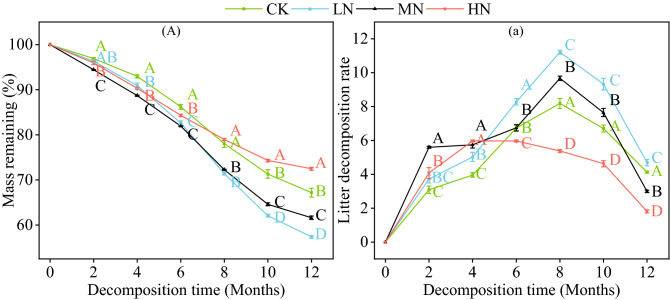
Dynamic changes of litter dry mass remaining (%) and litter decomposition rate during litter decomposition. Data show the mean ± SE (n = 3). Significant differences (*P* < 0.05) among treatments during a given sample time are indicated by different capital letters.

In this experiment, the litter quality declined throughout the experimental period (Fig. 1A). Within the first 6 months of decomposition, the litter lost 13.79% ~ 18.08% of its initial mass. The litter mass remaining in the CK treatment was significantly higher than in the other three treatments in the 4th and 6th month(*P* < 0.05, Fig. 1A). The remaining litter mass in the HN treatment was significantly higher than that in the CK, LN and MN treatments in the 10th and 12th month (*P* < 0.05). The LN and MN treatments were significantly lower than the CK treatment from the 6th month (*P* < 0.05). The decomposition rate of litter under the different treatments first accelerated and then decelerated (Fig. 1a). Among them, the decomposition rates of CK, LN and MN all peaked during the 8th month of the experiment and were 8.18, 11.20 and 9.67, respectively, whereas the decomposition rate of the HN treatment peaked during the 4th month at 5.98. The decomposition rates of LN in the 12th month were significantly higher than those in the CK group, and the decomposition rates of MN and HN were significantly lower than those in the CK group (*P* < 0.05).

### Effects of N addition on decomposition of lignin and cellulose in litter

**Figure 2:**
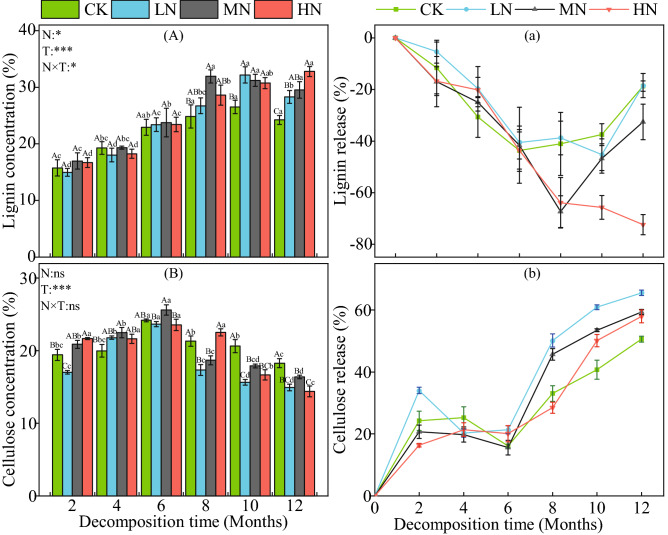
The dynamic changes of lignin and cellulose content and release rate during litter decomposition. Data show the mean ± SE (n = 3). N is N addition treatment and T is decomposition time; N × T: interaction between N addition and decomposition time. *P < 0.05; **P < 0.01; ***P < 0.001. Significant differences (*P* < 0.05) among sampling times are indicated by different lowercase letters and significant differences (*P* < 0.05) among treatments are indicated by different capital letters.

During the 12-months field litter decomposition experiment (Fig. 2A), the lignin content of the CK, LN and MN treatments showed an increasing then decreasing trend, while the lignin content in the HN treatment continued to increase monotonically during the decomposition period. The lignin content of the litter in all three N application treatments gradually increased while the lignin content in the control treatment decreased, with the divergence between CK and the other three treatments becoming statistically significant at the 8th month mark (*P* < 0.05). In all four treatments, the litter became enriched in lignin as a result of the decomposition process (Fig. 2a). In the HN treatment, lignin continued to be increasingly enriched throughout the decomposition period, while in the other three treatments lignin enrichment slowed during the 8th month and then started to exhibit a reduction in its relative concentration. The cellulose content of each treatment showed a similar nonlinear trend, first increasing in concentration before decreasing during the end of the decomposition period (Fig. 2B). The cellulose content peaked during the 6th month of litter decomposition. The cellulose content of the CK treatment was significantly higher than that of the three N treatments in the 12th month (*P* < 0.05). Cellulose was released during the 12-month decomposition test. The release rate was slower in the first 6 months of decomposition and gradually increased in the subsequent 6 months (Fig. 2b).

### Effects of N addition on litter C, N and P release

**Figure 3:**
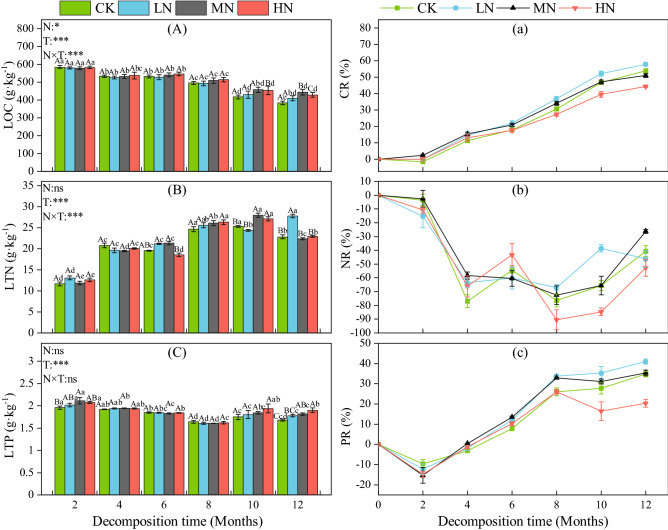
Dynamic changes of litter carbon, N, phosphorus content and release rate during litter decomposition. Data show the mean ± SE (n = 3). N is N addition treatment and T is decomposition time; N × T: interaction between N addition and decomposition time. *P < 0.05; **P < 0.01; ***P < 0.001. Significant differences (*P* < 0.05) among sampling times are indicated by different lowercase letters and significant differences (*P* < 0.05) among treatments are indicated by different capital letters.

The carbon content of litter (Fig. 3A) showed a downward trend throughout the decomposition period. There was no significant difference in carbon content between treatments within the first eight months of the experiment. Although the carbon content in the CK treatment during the last two sampling intervals was lower than in the N application treatments, the difference was not significant. The results of Repeated Measures ANOVA showed that N addition (*P* < 0.05) and decomposition time (*P* < 0.001) had a significant effect on the change in the carbon content of litter and that there was a significant interaction effect between the two (*P* < 0.001). Carbon in litter was released during the entire decomposition process (Fig. 3a), with the highest decomposition rate occurring at the end of decomposition in the LN treatment and the lowest decomposition rate was observed in the HN treatment. The change in N content (Fig. 3B) was nonlinear, first increasing gradually and then decreasing beginning at 10 months after the start of the experiment. Decomposition time had a significant effect on the N content of litter (*P* < 0.001), which was also significantly affected by the decomposition time and its interaction with N addition (*P* < 0.001). N was rapidly enriched within the first 4 months of the decomposition experiment, at which point the rate of enrichment slowed down and there was a trend of N release during the subsequent decomposition process (Fig. 3b). The phosphorus content in litter (Fig. 3C) showed a downward trend for the first eight months of the experiment and then a gradual increase. Decomposition time (*P* < 0.001) had a significant effect on the phosphorus content. There was no significant difference in the phosphorus content of the four treatments during the first eight months of the experiment. But, the phosphorus content of the CK treatment was significantly lower than that of the MN and HN treatments in the 12th month (*P* < 0.05). In the first two months of decomposition, the phosphorus content of the litter was enriched (Fig. 3c) but then was quickly released. After eight months of decomposition, the release rate began to gradually slow down.

### Effects of N addition on soil C, N, P, pH and enzyme activities

**Figure 4:**
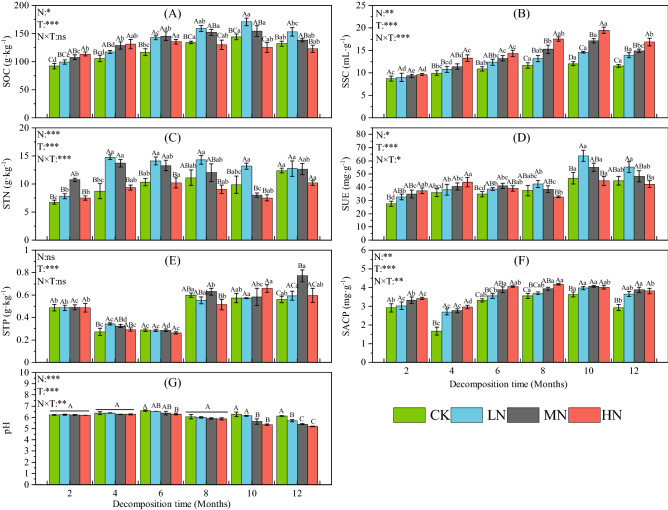
Dynamic changes of soil carbon, N and phosphorus content, pH and enzyme activity during litter decomposition. Data show the mean ± SE (n = 3). N is N addition treatment and T is decomposition time; N × T: interaction between N addition and decomposition time. *P < 0.05; **P < 0.01; ***P < 0.001. Significant differences (*P* < 0.05) among sampling times are indicated by different lowercase letters and significant differences (*P* < 0.05) among treatments are indicated by different capital letters.

Soil carbon (Fig. 4A), N (Fig. 4C) and phosphorus (Fig. 4E) concentration shows a showed nonlinear trend. The soil carbon content first increased and then decreased during the 12-month decomposition test. In the 4 months before decomposition, the soil carbon content increased with increasing N application, and in the subsequent decomposition process, the three N addition treatments, not including CK, showed the opposite trend. At the end of the experiment, the carbon content in the LN treatment was significantly higher than that CK and HN treatments (*P* < 0.05), and the carbon content in the HN treatment was the lowest. The soil N content first increased and then decreased after 10 months of decomposition. The N content in the LN and MN treatments was higher. By the end of the experiment, the N content of each treatment showed an increasing trend. The soil phosphorus content (Fig. 4E) decreased during the decomposition experiment but then increased by the 6th month. From the sixth month to the end of the experiment, the phosphorous content in the CK, LN and HN treatments did not change significantly. At the end of the experiment, MN had a significantly higher phosphorous content than the other three treatments. In the late decomposition period (10th and 12th month), N addition reduced soil pH (Fig. 4G). Specifically, the soil pH decreased significantly with an increasing nitrogen application rate. Nitrogen treatments and decomposition time had a significant effect on soil pH (*P* < 0.001), and the two had a significant interaction (*P* < 0.01).

The invertase activity in the soil (Fig. 4B) showed a slow upward trend during the decomposition process, but a downward trend occurred at the end of the experiment. The invertase activity increased with the amount of N in the different treatments. The decomposition process gradually showed significant differences between the treatments, and the addition of N significantly increased the invertase activity in the 8th, 10th, and 12th month (*P* < 0.05). The urease activity in soil (Fig. 4D) did not change significantly within 8 months before decomposition, but the urease activity increased significantly 4 months after decomposition. The phosphatase activity in the soil (Fig. 4F) decreased across all treatments during the 4th month of the experiment and then trended upwards before decreasing. The enzyme activity in the control treatment was lower than in the N application treatments in each sample, and the addition of N increased phosphatase activity.

### Relationship between litter decomposition characteristics and soil environment

**Figure 5:**
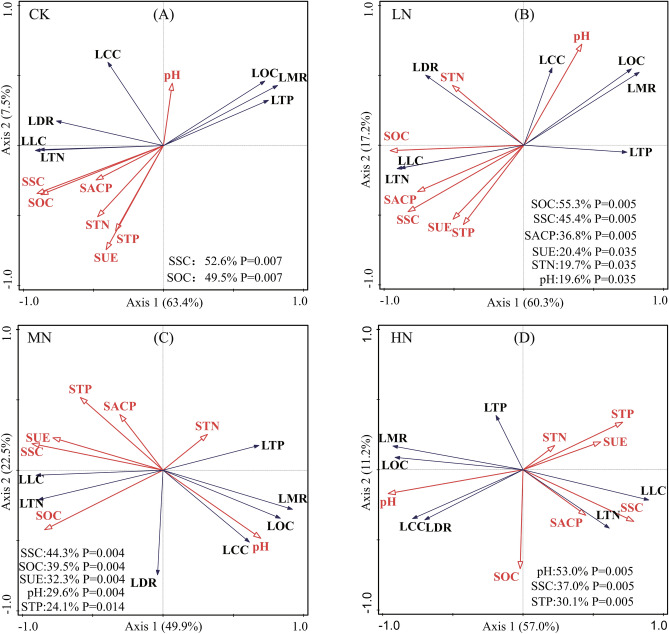
Two-dimensional graph of redundancy analysis of litter decomposition characteristics and soil variables at different N addition levels. Red hollow arrows represent soil environment and soil enzymes, and blue solid arrows represent litter decomposition characteristics. LMR is the litter mass remaining; LDR is the litter decomposition rate; LLC is the litter lignin content; LCC is the litter cellulose content; LOC is the litter organic carbon content; LTN is the litter N content; LTP is the phosphorus content of litter; SACP is the acid phosphatase activity; SSC is the sucrase activity, SUE is the urease activity; SOC is the soil organic carbon; STN is the soil total N content; STP is the soil total phosphorus content.

The RDA showed the effects of the soil environment and enzyme activity on litter decomposition characteristics (Fig. 5). The first and second axes explain 70.9%, 77.7%, 72.4% and 68.2% of the total variability in the litter decomposition characteristics under CK, LN, MN and HN treatments, respectively. This shows that the first two axes do a good job of capturing the relationship between the soil environment and litter decomposition characteristics. Correlation analysis of litter decomposition characteristics, soil nutrients, pH, and enzyme activity at different N levels was also conducted (Fig. S1). The analysis shows that in the CK and LN treatments, LDR was significantly positively correlated with LTN, LLC, SSC, and SOC and was significantly negatively correlated with LTP. In the MN treatment, LDR was only negatively correlated with LTP; in the HN treatment, LDR was positively correlated with LOC, LLC, and pH and negatively correlated with LTP. Soil pH was negatively correlated with SUE and SSC in the N application treatment, and the correlation increased with higher N application levels, and soil pH was negatively correlated with SACP in the LN treatment.

## Discussion

### Response of litter decomposition to N addition

Past studies have shown variable effects of N deposition on litter decomposition^21^. Our 1-year decomposition experiment showed that the N addition rate and decomposition time and their interaction are important factors that may help explain these varying findings. Specifically, the three N addition treatments promoted early-stage litter decomposition, while the HN treatment had a negative effect on the late stage of decomposition. This intriguing result may be due to regional differences in nitrogen deposition. N addition in a forest that is already saturated with N does not have a significant positive effect on the rate of litter decomposition, but it shows a significant positive effect in an N-limited forest^2,36^. The N deposition level in the middle of the Tianshan Mountains is below the average in China and the forest is characterized by high N deficiency^9,37^. In the case of moderate N deposition, exogenous N input can meet the needs of soil microorganisms for N and provide sufficient N resources for decomposers^38,39^. Microorganisms can be more active during the decomposition process and have a positive effect on litter decomposition^2^. Therefore, appropriate levels of N addition can have positive effects on litter decomposition in N-deficient pine forests and some temperate pine forests with low N^40^.

Many studies have shown that there is a threshold for the effect of N addition on litter decomposition. N application below this threshold may promote decomposition or have no effect^12^. However, when N application is higher than this threshold, it will inhibit the production of microbial decomposition enzymes and have a negative effect on litter decomposition^41^. Consistent with previous findings, there is an upper limit on the positive effect of N addition^42^. We found that the application of high N suppressed the decomposition of forest litter in the Tianshan Mountains. This discrepancy may be a result of a slightly higher N application in the highest N treatment group. Although our research emphasizes that the appropriate nitrogen input (LN and MN) has a positive effect on litter decomposition in the short-term for N-limited forests. However, to fully observe the response of litter decomposition to nitrogen input rate and decomposition time, the one-year decomposition test has limitations. Need to do more work on a complete litter decomposition process.

### Response of litter substrate to N addition

The degradation of lignin and cellulose influences the litter decomposition process and is therefore crucial for understanding litter decomposition dynamics. In this study, the addition of N promoted the degradation of cellulose but inhibited the degradation of lignin. The inhibitory effect of high N addition on lignin degradation was stronger than that of lower N addition. In previous studies, it has been reported that N addition promotes cellulose decomposition or causes no significant change, and most studies indicate that N addition inhibits lignin decomposition^16^. Cellulose is composed of long-chain glucose molecules with a relatively simple structure^43^. Generally, the addition of N will increase the activity of cellulase or cause no change^44^. Therefore, N addition makes cellulose easier to degrade. However, the litter of coniferous forests is usually characterized by a high content of lignin, which is composed of complex, stable and diverse amorphous three-dimensional macromolecules^43,45^, thus further slowing down the decomposition process. Increased N also decreases the production and activity of extracellular enzymes related to lignin degradation^46,47^. Excessive N may also combine with lignin and polyphenols to produce refractory compounds and increase the concentration of lignin^48^, which makes the degradation of litter with a high lignin content more strongly inhibited by N deposition.

The accumulation and release of C, N and P during litter decomposition is an important process in nutrient cycling in forest ecosystems. The increase in N deposition changes the soil’s N availability and the microbial decomposition process^2^ and significantly affects the dynamic change in litter nutrients. Our research shows that carbon and phosphorus were released during litter decomposition, and N first accumulated and then was released. High N addition had a negative effect on the release of carbon and phosphorus, while low N addition had a more notable stimulating effect. In N-deficient forest ecosystems, exogenous N input significantly affects the growth of decomposers (microorganisms and enzymes) and shifts the soil nutrient balance^41^. However, an appropriate amount of N input relieves the demand by decomposers for N and promotes the use of nutrients in the litter by the decomposers^45^. Therefore, low N input is conducive to the release of carbon and phosphorus in litter. However, as the amount of N increases, the N in the environment gradually reaches saturation, where high N addition gradually shows an inhibitory effect^49^.

### Response of soil environment to N addition

Increasing N deposition increases available N in the soil, as well as the utilization of nutrients by soil microorganisms and the activity of soil enzymes changes^24,27^. Soil enzymes are directly involved in litter decomposition and nutrient cycling and play an important role in soil C, N and P cycling^46^. Previous studies have reported that N addition can have a positive, negative, or no effect on soil enzyme activity^41,50^. Our results showed that soil enzyme activity, especially sucrase activity, increased significantly after N application. Sucrase can directly participate in the metabolism of soil organic matter containing carbon, is responsible for the release of low molecular weight sugar, and plays a key role in litter decomposition in each treatment (Fig. 5). Litter provides abundant carbon sources for the soil environment in N-poor forest ecosystems^51^. However, N gradually becomes an important limiting factor for sucrase activity, which can be compensated for by exogenous N input. The addition of N may increase the microbial demand for phosphorus, thus increasing phosphorus-related enzymes in the soil^52,53^.

In addition, it is worth noting that urease activity responds differently to different levels of N addition at different stages of decomposition. Enzyme activity rises with increasing levels of N addition during the early stage of decomposition but shows the opposite trend during the late stages of decomposition. Exogenous N input may increase the N demand of microorganisms by increasing the effectiveness of soil N, thereby increasing the activity of enzymes related to the soil N cycle (such as urease)^54^. However, high levels of N addition can saturate and then exceed the demand for N, leading to high NH_4_^+^ contents in the soil, causing toxicity or inhibition to the soil microbial community, thus reducing enzyme activity^55^. Previous studies indicate that soil acidification caused by N addition can inhibit soil enzymes^56^. Previous studies pointed to N addition decreases soil pH and significantly decreases with increasing N addition^25,57,58^. Our results show that N treatments (from HN to LN) begin to significantly decrease soil pH during the 10th and 12th months of decomposition. This may be due to leaching of NO_3_^−^ and reduction of basic cations with increasing N application^59^. Although previous studies pointed to the effect of long-term N addition on soil pH^25,59^. However, few studies have pointed out the critical point of soil pH change (nitrogen application rate and experiment time), which is more important. Our results may be able to provide some information. In addition, our conclusions show that soil acidification caused by N addition does not inhibit soil enzymes. We speculate that the soil enzymes in the study area have been in an acidic environment for a long time and have developed strong adaptability. N addition did not reach the critical value of inhibiting enzyme activity.

In this study, we also found that in the 2th and 4th month, N addition increased the soil carbon content, and it increased with increasing in nitrogen application rate. However, it showed the opposite pattern in the 6th, 8th, 10th, and 12th month. In the decomposition process, soil nutrients are affected by litter decomposition characteristics (such as litter mass loss and nutrient release) and soil enzyme activity^46^. Some studies shown that early-stage acceleration litter decomposition increases the release of DOC from litter pools, and can be stabilized on chemical and physical interactions with soil minerals^60,61^. A new studies evidence that N addition can increase soil carbon stocks by reducing CO_2_ emissions and litter decomposition in N-limited ecosystems^62^. Furthermore, microbial necromass is an important source of soil organic carbon^63^. Some studies point to a general increase in microbial in N-limited ecosystems^64^.

## Conclusions

In summary, the simulated N deposition experiment significantly affected the decomposition of litter and the soil environment in the *Picea schrenkiana* forest of the Tianshan Mountains, China. When the rate of ambient N deposition is low then the main factor that determines the degree and direction of the impact is the N application rate. The LN treatment had a positive effect on litter decomposition and the soil environment, while the HN treatment significantly increased the remaining litter mass and lignin content. N addition caused soil acidification, but it did not negatively affect soil enzyme activities. N addition significantly increased invertase activity and phosphatase activity. Meanwhile, RDA showed that enzyme activity had a greater contribution to litter decomposition, especially in the LN treatment. The results of the present study can help researchers better understand the response of litter decomposition and the soil environment to increased N deposition in N-limited forest ecosystems in the context of global change. And highlight the importance of long-term measurements for accurately assessing the effects of N inputs on litter decomposition and soil. Short-term nitrogen additions cannot provide complete information, for example soil acidification and carbon dynamics.

## Materials and methods

### Site description

This study was conducted in a forest ecosystem in the middle of the Tianshan Mountains (42° ~ 45°N, 83° ~ 94°E; 1945 m.a.s.l.), Xinjiang, China. The study site has a temperate continental climate. The mean annual precipitation is approximately 500 mm. The mean annual temperature is approximately 2.5 °C, and the annual total radiation is approximately 5.85 × 105 J cm^−2^ a^−1^. The main forest type is classified as a northern temperate coniferous forest, and *Picea schrenkiana* (*Picea schrenkiana* Fisch. & CA. May, Pinaceae) is the dominant species. The soil is mainly taupe forest soil which has developed over calcium rock differentiation material, and is weakly acidic.

### Experimental design and sampling

In September 2017, a total of 12 plots were established in a representative evergreen coniferous forest site. Each plot was 2 m × 2 m and characterized by the same altitude, slope, aspect, and forest age. There was a 5 m buffer zone between each plot, and surface litter removal occurred simultaneously at each plot. Four treatment levels were set for this study according to the local nitrogen deposition background value level (5 kg N ha^−1^ a^−1^): control (CK; no N addition), low N addition (LN; 5 kg N ha^−1^ a^−1^), medium N addition (MN; 10 kg N ha^−1^ a^−1^), and high N addition (HN; 20 kg N ha^−1^ a^−1^). Three replicates were established for each treatment level. Urea solution was applied every two months from October 2017 to October 2018. The urea required for each treatment level and each N application was calculated and dissolved in 500 mL of deionized water. Then, it was sprayed evenly back and forth at a height of 50 cm above the ground for each plot. The control plot received 500 mL deionized water.

In September 2017, a square litter collector (with a surface area of 1 m^2^) was used to collect fresh litter (only leaves). Fallen leaves were separated from other debris and brought to a laboratory for natural air-drying. During the air-drying process, the fallen leaves were fully mixed to ensure that the litter was homogenous in the litter bags. Ten grams of air-dried litter was weighed and put into 360 (12 plots × 6 samples × 5 litter bags) litter bags (20 cm × 20 cm, bottom aperture: 0.5 mm, top aperture: 2.0 mm). Five decomposition bags were randomly selected and dried at 65 °C until the sample weight remained constant to determine the initial chemical properties of the litter: lignin content (LLC) was 13.96%, cellulose content (LCC) was 24.85%, total carbon content (LOC) was 557.9 g kg^−1^, total N content (LTN) was 10.91 g kg^−1^, and total phosphorus content (LTP) was 1.73 g kg^−1^. In October 2017, the litter bags were evenly arranged on the surface of each sample plot and fixed with nylon rope.

Three litter bags were randomly selected from each sample every two months from the start of N application (a total of six samples in December 2017 and February, April, June, August, and October in 2018). The litter bags were taken back to the laboratory, debris was removed, and the bags were cleaned with distilled water. A 75 °C oven was used to dry the samples until they achieved a constant weight, and the dry weighed litter was used to determine the LOC, LTN, LTP, LLC and LCC. The soil samples were sampled when we taken the litter bags. In each plot, five soil samples from the 0–20 cm soil layer were taken with a soil auger (Φ = 5 cm) according to the “S” method after removing the litter layer. After the debris was removed, it was divided into two parts: one part was stored at − 20 °C for measuring soil enzyme activity, and the other part was air-dried to measure soil pH, soil organic carbon (SOC), total N (STN) and total phosphorus (STP).

### Chemical analysis

The total C concentration in the litter was measured by a potassium dichromate oxidation titration with a solution of Fe^2+^^65^. The total N concentration in the litter was determined through acid digestion using the Kjeldahl method^66^. The total P concentration was analyzed by the molybdenum-antimony colorimetric method after the samples were digested with H_2_SO_4_^67^. The lignin and cellulose concentrations were determined using the acid detergent fiber method^68^. The total C, N, and P concentrations in the soil were determined with the same method as that in the litter but with some modifications. The soil pH was measured in a soil–water suspension (1:2.5 v:v) with a glass electrode (PHS-3E, Leici, Shanghai). The methods described for the determination of soil enzyme activity were in (Kotroczó et al., Paolo et al., Schinner and Mersi)^69–71^. The urease activity of the soil was measured by indophenol colorimetry with urea as the substrate. The quantity of ammonium released over 24 h was colorimetrically assayed at 578 nm and expressed as a mg g^−1^ dry sample at 24 h. The invertase activity of the soil was assessed using 1.2% sucrose as the substrate, with an incubation period of 24 h at 37 °C, and expressed as mg g^−1^ dry sample at 24 h. The acid phosphatase activity of the soil (SACP) is based on the release of pNP from p-nitrophenyl-phosphate and is terminated using 0.5 mL of 0.5 M CaCl_2_ and 2 mL of 0.5 M NaOH. The activities were expressed as mg pNP released g^−1^ dry soil h^−1^. All enzyme activity determinations were performed in triplicate and averaged for further analysis.

### Data analysis

The remaining litter mass (LMR), litter nutrients (C, N, P), cellulose and lignin release rate (R, %) were calculated using the following formulas:

$${\text{LMR}} = \left( {{{{\text{M}}_{{\text{t}}} } \mathord{\left/ {\vphantom {{{\text{M}}_{{\text{t}}} } {{\text{M}}_{{\text{o}}} }}} \right. \kern-\nulldelimiterspace} {{\text{M}}_{{\text{o}}} }}} \right) \times 100\%$$

$${\text{R}}(\% ) = {{\left( {{\text{M}}_{{\text{o}}} {\text{C}}_{{\text{o}}} - {\text{M}}_{{\text{t}}} {\text{C}}_{{\text{t}}} } \right)} \mathord{\left/ {\vphantom {{\left( {{\text{M}}_{{\text{o}}} {\text{C}}_{{\text{o}}} - {\text{M}}_{{\text{t}}} {\text{C}}_{{\text{t}}} } \right)} {{\text{M}}_{{\text{o}}} {\text{C}}_{{\text{o}}} }}} \right. \kern-\nulldelimiterspace} {{\text{M}}_{{\text{o}}} {\text{C}}_{{\text{o}}} }} \times 100\%$$

where M_t_ is the mass of litter at time t (g), M_o_ is the initial dry weight of litter (g), C_o_ is the initial content of nutrients, lignin, and cellulose in the litter, and C_t_ is the concentration at time t.

We used SPSS 25.0 (SPSS Inc., Chicago, USA) for all statistical analyses, taking p < 0.05 as an indicator of statistical significance. Repeated Measures ANOVA was used to analyze the effects of fertilizer concentration and fertilization frequency on lignin and cellulose concentration, litter, and soil nutrient elements (C, N, P), and soil enzyme activities (sucrose, urease, phosphatase). The least significant difference multiple comparison (Fisher’s LSD) test was used to analyze the difference in treatment and release time of lignin and cellulose concentration, litter, and soil nutrient elements (C, N, P), and soil enzyme activity. Redundancy analysis (RDA) was used to determine which environmental factors were related to the litter decomposition process. The most distinctive environmental factors were selected through the program's "positive selection" process. Positive selection is based on Monte Carlo sorting (n = 499), using Canoco 5.0 for Windows (Ithaca, NY, USA) to run the statistical significance test of RDA. Pearson correlation analysis was performed to evaluate the relationships between litter decomposition and soil environmental factors. The correlation analysis was performed in R 3.5.2 (R Development Core Team, 2015). Origin Pro 2019b (Origin Lab. Inc., Massachusetts, USA) was used for creating the figures.

## Supplementary Information

Supplementary Information.
