# Supplementary material for: Response of litter decomposition and the soil environment to one-year nitrogen addition in a Schrenk spruce forest in the Tianshan Mountains, China
Source: Sci Rep. 2022 Jan 13;12:648. doi: 10.1038/s41598-021-04623-8 (PMC8758753; doi:10.1038/s41598-021-04623-8)
Supplement: Supplementary file 1 — Supplementary Information. [file 41598_2021_4623_MOESM1_ESM.docx]

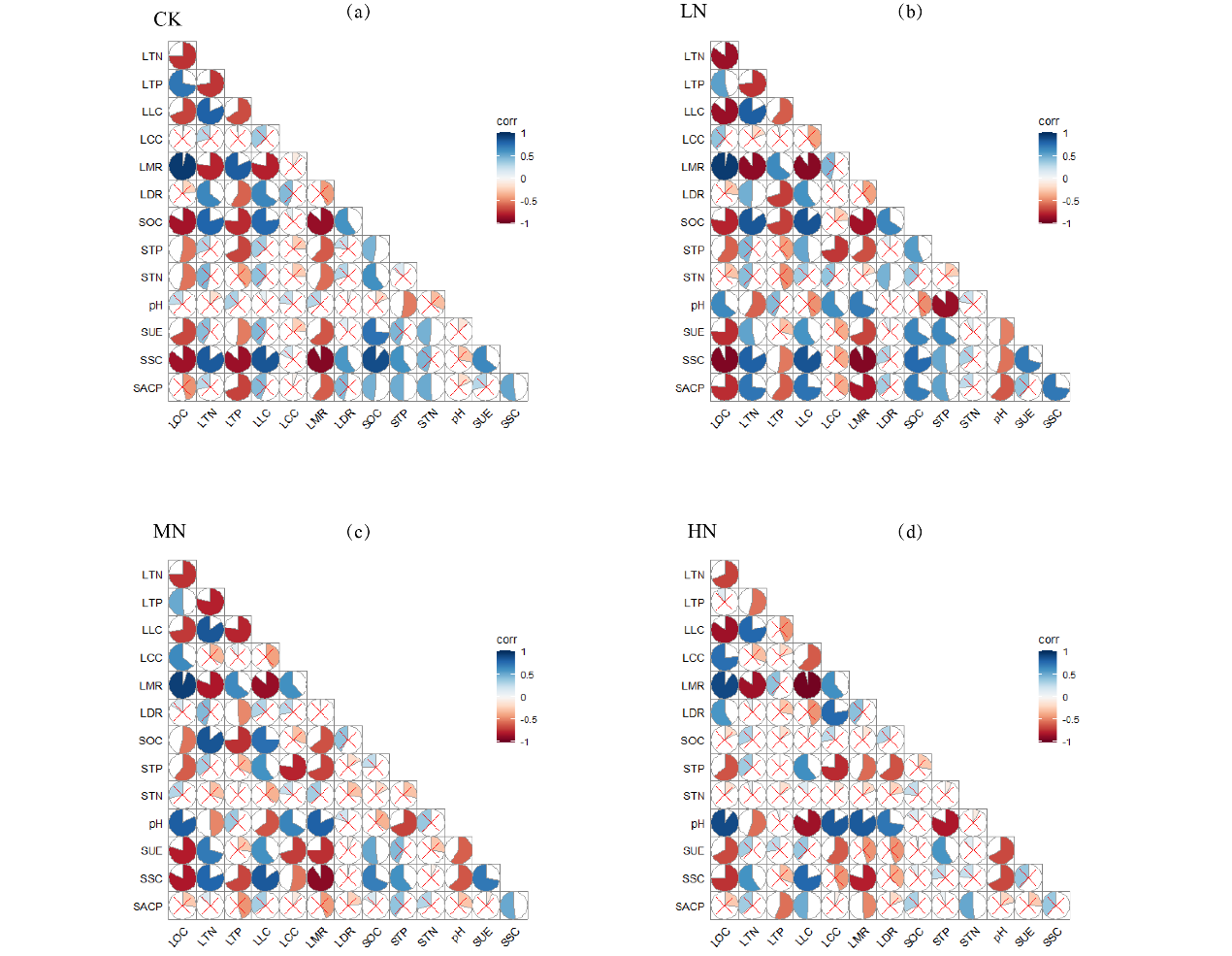
**Fig. S1**

**Fig. S1** Correlation analysis of litter decomposition characteristics and soil variables at different N addition levels. LMR is the litter mass remaining; LDR is the litter decomposition rate; LLC is the litter lignin content; LCC is the litter cellulose content; LOC is the litter organic carbon content; LTN is the litter N content; LTP is the phosphorus content of litter; SACP is the acid phosphatase activity; SSC is the sucrase activity, SUE is the urease activity; SOC is the soil organic carbon; STN is the soil total N content; STP is the soil total phosphorus content.
